# Evaluation of additional gastrectomy after noncurative endoscopic submucosal dissection for early gastric cancer

**DOI:** 10.1186/s12893-022-01777-8

**Published:** 2022-10-01

**Authors:** Shinichiro Makimoto, Yutaka Mushiake, Tomoya Takami, Hiroshi Shintani, Naoki Kataoka, Tomoyuki Yamaguchi, Shoji Oura

**Affiliations:** Department of Surgery, Kishiwada Tokushukai Hospital, 4-27-1 Kamori-Cho, Kishiwada, Osaka 596-8522 Japan

**Keywords:** Endoscopic submucosal dissection, Early gastric cancer, Additional gastrectomy, Lymph node metastasis, Local residual cancer

## Abstract

**Background:**

Performing additional surgery after noncurative endoscopic submucosal dissection (ESD) for early gastric cancer is controversial. Our aims are to clarify the risk factors for lymph node metastasis (LNM) and local residual cancer (RC) after noncurative ESD and to determine recommendations for additional treatment.

**Methods:**

Of the 1483 patients who underwent ESD for early gastric cancer between January 2012 and April 2020, we retrospectively analyzed 151 patients diagnosed as having a lesion not meeting the curative criteria after ESD. Of these patients, 100 underwent additional gastrectomy, and 51 were observed without surgery.

**Results:**

Surgical specimens showed LNM in 14 patients (14.0%) and local RC in 7 (7.0%). However, 81 patients (81.0%) had neither of these malignancies. Multivariate analysis revealed that a positive lymphatic invasion (*P* = 0.035) and an undifferentiated type (*P* = 0.047) were independent risk factors for LNM, whereas a positive horizontal margin (*P* = 0.010) was an independent risk factor for local RC. Furthermore, the prevalence of LNM was significantly higher in patients with both positive lymphatic and vascular invasions. In the additional gastrectomy group, 3 patients (3.0%) had recurrences, and 2 patients (2.0%) who had distant recurrences died of gastric cancer. In the observation group, recurrence was observed in 3 patients (5.9%). One patient (2.0%) who had liver metastasis died of gastric cancer. Of the 2 patients (3.9%) who had local recurrences, one underwent additional ESD, and the other without additional ESD died of other disease.

The 5-year overall survival rates in the additional gastrectomy and observation groups were 87.4% and 73.8%, respectively (log-rank test, *P* = 0.008).

**Conclusion:**

Following noncurative ESD for early gastric cancer, we recommend an additional gastrectomy with lymph node dissection for patients with lymphovascular invasion and/or undifferentiated type. Careful follow-ups without additional surgery may be acceptable for patients with advanced age, severe comorbidity, or no lymphovascular invasion.

## Introduction

Endoscopic submucosal dissection (ESD) is a standard treatment for early gastric cancer with a negligible risk of lymph node metastasis [1, 2]. According to Japanese gastric cancer treatment guidelines, resection of a lesion that does not meet the curative criteria is considered a noncurative resection and radical gastrectomy with lymph node dissection or additional endoscopic treatment is the recommended course of treatment [1–3]. In general, it is difficult to determine if there are patients with clinically positive nodes after ESD. Therefore, the necessity for additional surgery is determined based on the histopathologic findings of the endoscopically resected specimens [3]. If positive horizontal margin is the only non curative factor, additional ESD is considered [1, 2]. Especially for elderly patients and those with complications, an additional gastrectomy is invasive and potentially fatal. Therefore, whether an additional gastrectomy is necessary for all patients not meeting the curative criteria after ESD is controversial [4, 5].

In this study, we evaluated the predictive factors for LNM and local residual cancer (RC) in patients not meeting the curative criteria after ESD and, based on these findings, recommend criteria to determine additional treatment strategies.

## Patients and methods

A total 1483 patients underwent ESD for early gastric cancer at the Department of Gastroenterology at Kishiwada Tokushukai Hospital between January 2012 and April 2020. Of these, we analyzed 151 patients diagnosed as having a lesion not meeting the curative criteria after ESD, including 100 who underwent additional gastrectomy, and 51 who were observed without surgery. All cases of ESD were en bloc resection; piecemeal resection was not included in these cases.

This study was approved by the institutional ethics committee of Kishiwada Tokushukai Hospital.

### Curative criteria

Tumors were classified by invasion depth as either mucosal (M) or submucosal (SM). Submucosal cancers were subclassified, according to the depth of the tumor invasion into the submucosa measured from the muscularis mucosae, as either superficial (SM1; depth < 500 *µm*), or deep (SM2; depth ≥ 500 *µm*) submucosal invasion. Results of histopathological examination of the ESD specimen were evaluated according to the Japanese classification of gastric carcinoma [1, 3] for curative criteria based on several indicators.

Curative resection was indicated for a case of an en bloc resection with 6 coexisting indicators, including (i) a tumor size ≤ 2 cm, (ii) differentiated tumor type, (iii) pT1a (M), (iv) negative horizontal margin (HM0), (v) negative vertical margin (VM0), and (vi) no lymphovascular invasion (ly[−], v[−]).

Curative resection for tumors of expanded indication was defined for an en bloc resection with 3 indicators, including (i) HM0, (ii) VM0, (iii) ly(−), v(−), and (iv) either of four types of constellation of an additional set indicators, including (1) a tumor size > 2 cm, differentiated tumor type, pT1a, with a negative ulcerative finding (UL[−]); (2) a tumor size ≤ 3 cm, differentiated tumor type, pT1a, and UL( +); (3) a tumor size ≤ 2 cm, an undifferentiated tumor type, pT1a, and UL(−); or (4) a tumor size ≤ 3 cm, differentiated tumor type, and pT1b (SM1) [3].

Noncurative resection was the resection of a lesion that does not meet any of the above curative criteria [3]. Whether additional gastrectomy or observation was finally chosen depended on patient’s age, comorbidities, and decision.

### Pathological evaluation

Following resection, ESD specimens were immediately fixed in 10% buffered formalin, serially sectioned to 2 mm thickness, and the sections mounted on slides were stained with hematoxylin and eosin. Stained sections were examined by a pathologist, and a pathological diagnosis was made and the depth of tumor invasion was measured according to the Japanese classification of gastric carcinoma [1]. Lymphatic invasion and vascular invasion were assessed by D2-40 staining and Elastica van Gieson staining, respectively.

### Additional surgery and follow-up

Following noncurative ESD, patients were evaluated for additional surgery with lymph node dissection. In principle, a radical gastrectomy with concomitant lymph node dissection is recommended after noncurative ESD [1, 3]. The decision on the type of gastrectomy is decided, according to the Japanese gastric cancer treatment guidelines [1].

Distal gastrectomy is selected, when proximal distance from the cardia is 2 cm or more. Total gastrectomy is selected when proximal distance from the cardia is < 2 cm. Proximal gastrectomy is selected for proximal tumors, where more than half of the distal stomach can be preserved. In our institution, a lymph node dissection is performed with D1 + as standard treatment. D2 lymph node dissection was performed in patients whose ESD specimens showed SM2 invasion with a positive vertical margin or who had clinical suspicion of LNM. With the patient’s informed consent, either open or laparoscopic surgery was performed. After the additional gastrectomy, we planned to follow up the patients for at least 5 years, with abdominal computed tomography (CT) and ultrasonography performed every 6 months to monitor distant metastases.

For patients who did not undergo additional gastrectomy, surveillance endoscopy was performed following noncurative ESD at every 6 months in the first two years, and annually later. Abdominal CT was performed every 6 months to monitor LNM and distant metastases.

### Statistical analysis

Differences of means between categorical variables were analyzed using Fisher’s exact test. Odds ratios and 95% confidence intervals were calculated to evaluate the correlation of LNM and RC with each risk factor. We selected the variables that appeared to be relevant to outcomes in multivariate analysis, based on the findings of previous studies and clinical judgement. We used multivariate logistic analysis to identify the risk factors for RC using the factors of tumor size, depth of invasion, ulceration, lymphatic invasion, horizontal margin, and vertical margin, and LNM using factors of histological type and vascular invasion in addition to the six factors for RC. Overall survival and disease-specific survival were calculated according to the Kaplan–Meier method and were analyzed by the log-rank test. Statistical significance was indicated by *P* value < 0.05. All statistical analyses were performed using R version 3.3.2 (The R Foundation for Statistical Computing, Vienna, Austria).

## Results

**Fig. 1:**
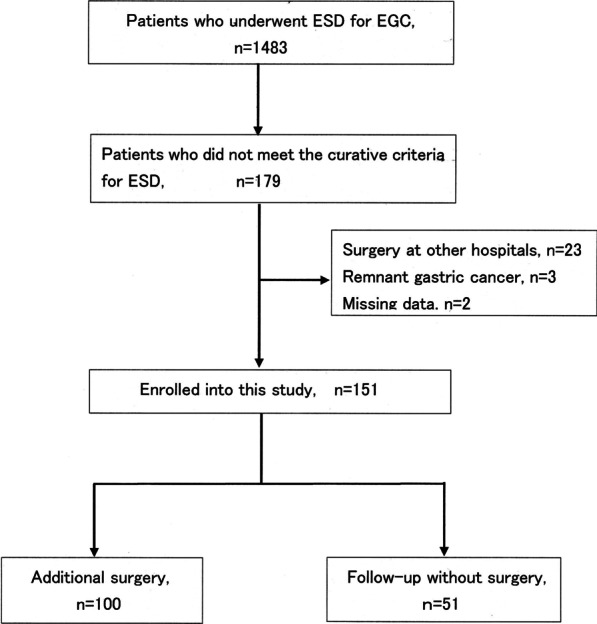
Flowchart of patient enrollment. *EGC* early gastric cancer, *ESD* endoscopic submucosal dissection

We retrospectively analyzed data from 1483 patients who underwent ESD for early gastric cancer at our hospital over an 8-year period, and 179 patients were diagnosed as not meeting the curative criteria after ESD (Fig. 1). Of the 179 patients, 23 patients underwent surgery at other hospitals, 3 patients of remnant gastric cancer, and 2 patients with missing data, were excluded from this study. Of the remaining 151 patients, 100 patients underwent additional gastrectomy, and 51 patients were observed without surgery.

The reasons for no additional gastrectomy after non curative ESD in the observation group were as follows: 11 patients with advanced age ≥ 80 years, 13 patients with severe comorbidities, and 25 patients with patients’ decision.The remaining 2 patients did not have the gastrectomy because of undergoing additional ESD.

The clinicopathological characteristics of the additional gastrectomy and observation groups are shown in Table 1. In the observation group, the patients’ clinicopathological characteristics did not differ significantly from additional gastrectomy group, but age (*P* < 0.001) and American Society of Anesthesiologists physical status (ASA-PS) (*P* = 0.027) were significantly high. There was no difference in tumor size and histopathological type between the two groups. The cancer depth in the ESD specimens in the additional gastrectomy group indicated invasion class M in 11 patients, SM1 in 9 patients, SM2 in 78 patients, and musculus propria (MP) in 2 patients. On the other hand, the cancer depth in the observation group indicated invasion class M in 6 patients, SM1 in 2 patients and SM2 in 43 patients. The rates of positive lymphatic invasion and positive vertical margin tended to increase in the additional gastrectomy group compared to the observation group.

**Table 1:** Clinicopathological characteristics of patients in the additional gastrectomy and observation groups after noncurative ESD

	Additional gastrectomy		Observation		*P*
	n = 100	%	n = 51	%	
Age, median, range (years)	70 (38–87)		77 (53–94)		< 0.001
Sex					0.337
Male	76	76	35	68.6	
Female	24	24	16	31.4	
ASA-PS					0.027
I	5	5	0	0	
II	89	89	42	82.4	
III	6	6	9	17.6	
Tumor location					0.545
Upper	33	33	14	27.4	
Middle	28	28	19	37.3	
Lower	39	39	18	35.3	
Tumor size, median, range(mm)	24 (8–95)		22 (5–110)		0.531
Histopathological type					0.499
Differentiated	81	81	44	86.3	
Undifferentiated	19	19	7	13.7	
Ulceration					
Negative	82	82	46	90.2	0.235
Positive	18	18	5	9.8	
Depth of invasion					0.606
M	11	11	6	11.8	
SM1	9	9	2	3.9	
SM2	78	78	43	84.3	
MP	2	2	0	0	
Lymphatic invasion					0.078
Negative	57	57	37	72.5	
Positive	43	43	14	27.5	
Vascular invasion					0.839
Negative	78	78	39	76.5	
Positive	22	22	12	23.5	
Horizontal margin					
Negative	94	94	49	96.1	0.718
Positive	6	6	2	3.9	
Vertical margin					
Negative	81	81	47	92.2	0.094
Positive	19	19	4	7.8	

*ESD:* endoscopic submucosal dissection, *ASA-PS* American Society of Anesthesiologists physical status

*M* mucosa, *SM* submucosa, *MP* musculus propria

We performed distal gastrectomy in 60 patients, total gastrectomy in 29 patients, and proximal gastrectomy in 11 patients (Table 2). Most of the operations (89.0%; 89/100) were laparoscopic surgery. Lymph node dissection was performed with D1 + as standard treatment. LNM was observed in 14 patients (14.0%). Half of the 14 patients with LNM had 2 or more metastasized lymph nodes, including the 3 patients who recurred after the additional surgery. Local RC was observed in 7 patients (7.0%).

**Table 2:** Operations and postoperative outcomes

	n = 100	%
Surgery (open/laparoscopic)		
Distal gastrectomy	60 (3/57)	60
Total gastrectomy	29 (4/25)	29
Proximal gastrectomy	11 (4/7)	11
Operation time, median (IQR), minutes	300 (255–363)	
Extend of lymph node dissection		
D1	10	10
D1 +	78	78
D2	12	12
Number of retrieved lymph nodes, median (IQR)	29 (21–39)	
Status of residual cancer		
LNM	14	14
Local RC	7	7
Postoperative complications		
Anastomotic leakage	3	3
Intraabdominal abscess	3	3
Stomach stagnation	3	3
Pancreatic fistula	2	2
Pneumonia	1	1
SSI	1	1
Death	1	1
Hospital stay, median, range (days)	11 (6–109)	
Postoperative long-term outcome		
Recurrence	3	3
Death from gastric cancer	2	2

*IQR* interquartile range, *LNM* lymph node metastasis, *RC* residual cancer

*SSI* surgical site infection

Postoperative complications classified as Clavien-Dindo (CD) grade II or more were observed in 14 patients (14.0%), including anastomotic leakage in 3 patients, intraabdominal abscess in 3 patients, and pancreatic fistula in 2 patients. The median age of patients with postoperative complications grade II or more CD classification was 75 years. Of the 4 patients (4.0%) who suffered from grade III or more complications according to the CD classification, the two patients underwent surgical treatment for anastomotic leakage, the other one underwent drainage for an intraabdominal abscess, and they were discharged without any problem, but the remaining one died from aspiration pneumonia 51 days after surgery. The median postoperative hospital stay was 11 days.

The results of risk factor analysis of the 14 patients (14.0%) with LNM are shown in Table 3. Among the 14 patients with LNM, a lymphatic invasion was observed in 12 patients, and vascular invasion was observed in 7 patients. Univariate analysis identified significant correlations with LNM for both a positive lymphatic invasion (*P* < 0.001) and positive vascular invasion (*P* = 0.012). In contrast, tumor size, depth of invasion, and histological type were not correlated with LNM. Multivariate factor analysis confirmed the significant correlation of a positive lymphatic invasion (*P* = 0.035) and an undifferentiated type (*P* = 0.047) with LNM. Although a positive vascular invasion tended to be associated with LNM, the association was not statistically significant. In addition, relationship between lymphatic invasion and vascular invasion for LNM are shown in Table 4. LNM was observed in 7 patients (41.2%, 7/17) with both lymphatic and vascular invasions, and 2 patients (3.8%, 2/52) without both lymphatic and vascular invasions, respectively. The prevalence of LNM was significantly higher in patients with both positive lymphatic and vascular invasions.

**Table 3:** Risk factors for lymph node metastasis

	LNM Positive	LNM Negative	Univariate	Multivariate	
	(n = 14)	(n = 86)	*P*-value	OR (95% CI)	*P*
Tumor size					
≤ 30 mm	7	57		Reference	
> 30 mm	7	29	0.248	2.51 (0.58–10.80)	0.217
Depth of invasion					
M-SM1	0	20			
SM2 or deeper	14	66	0.066	NS	
Histological type					
Differentiated	10	71		Reference	
Undifferentiated	4	15	0.460	8.39 (1.03–68.20)	0.047
Ulceration					
Negative	9	73		Reference	
Positive	5	13	0.125	3.97 (0.74–21.50)	0.109
Lymphatic invasion					
Negative	2	55		Reference	
Positive	12	31	< 0.001	7.61 (1.15–50.30)	0.035
Vascular invasion					
Negative	7	71		Reference	
Positive	7	15	0.012	3.41 (0.73–15.90)	0.119
Horizontal margin					
Negative	13	81			
Positive	1	5	1.000		
Vertical margin					
Negative	10	71			
Positive	4	15	0.460		

*LNM* lymph node metastasis, *M* confined to mucosa, *SM1* depth of invasion from the muscularis mucosa < 500 *µm*

*SM2* depth of invasion from the muscularis mucosa ≥ 500 *µm*, *OR* odds ratio, *CI* confidence interval, *NS* not significant

**Table 4:** Relationship between lymphatic invasion and vascular invasion for lymph node metastasis

	LNM Positive		LNM Negative		Total
	n = 14	%	n = 86	%	n = 100
Ly (** +**) and V (** +**)	7	41.2	10	58.8	17
Ly (** +**) and V ( −)	5	19.2	21	80.8	26
Ly ( −) and V (** +**)	0	0	5	100	5
Ly ( −) and V (-)	2	3.8	50	96.2	52

*LNM* lymph node metastasis, *Ly(* +*)* positive lymphatic invasion, *Ly( −)* negative lymphatic invasion,

*V(* +*)* positive vascular invasion, *V( −)* negative vascular invasion

The risk factors for local RC in surgical specimens are shown in Table 5. Local RC was observed in 7 patients (7.0%). According to histopathological examination of the ESD specimens from these 7 patients, 3 patients had a positive horizontal margin, 3 patients had a positive vertical margin, and 1 patient had a negative margin. The pathological examination of the patient with the negative margin showed a tumor size of 76 mm with ulceration and positive lymphatic invasion. Univariate analysis identified significant correlations with Local RC for a positive horizontal margin (*P* = 0.004), tumor size > 30 mm (*P* = 0.008), and positive ulceration (*P* = 0.019). Although, multivariate factor analysis indicated that having local RC was significantly correlated with only positive horizontal margin (*P* = 0.010).

**Table 5:** Risk factors for local residual cancer

	Local RC Positive	Local RC Negative	Univariate	Multivariate	
	(n = 7)	(n = 93)	*P*-Value	OR (95% CI)	*P*
Tumor size					
≤ 30 mm	1	63		Reference	
> 30 mm	6	30	0.008	176.00 (0.92–33,800.00)	0.054
Depth of invasion					
M-SM1	2	18			
SM2 or deeper	5	75	0.625		
Histological type					
Differentiated	6	75			
Undifferentiated	1	18	1.000		
Ulceration					
Negative	3	79		Reference	
Positive	4	14	0.019	16.00 (0.99–259.00)	0.051
Lymphatic invasion					
Negative	2	55		Reference	
Positive	5	38	0.136	22.80 (0.61–860.00)	0.091
Vascular invasion					
Negative	6	72			
Positive	1	21	1.000		
Horizontal margin					
Negative	4	90		Reference	
Positive	3	3	0.004	71.30 (2.83–1790.00)	0.010
Vertical margin					
Negative	4	77		Reference	
Positive	3	16	0.124	21.50 (0.82–564.00)	0.066

*RC* residual cancer, *M* confined to mucosa, *SM1* depth of invasion from the muscularis mucosa < 500 *µm*

*SM2* depth of invasion from the muscularis mucosa ≥ 500 *µm*, *OR* odds ratio, *CI* confidence interval

The median follow-up period in the additional surgery group was 44 months (interquartile range [IQR], 20.5–65.5 months). During follow-up, 3 patients (3.0%) had recurrences: 1 each in the peritoneum, bone, and lymph nodes (Table 2). Two patients (2.0%) died of gastric cancer, and 6 patients (6.0%) died of other diseases.

Of the 51 patients in the observation group, 1 patient (2.0%) died as a result of gastric cancer, and 10 patients (19.6%) died from other diseases. The median follow up period in the observation group was 31 months (IQR 16.0–47.8 months), shorter than in the gastrectomy group. Recurrence was observed in 3 patients (5.9%). One patient (2.0%) who had liver metastasis died of gastric cancer. Of the two patients (3.9%) who had local recurrences, one underwent additional ESD, and is alive without recurrence, and the other without additional ESD died of myocardial infarction. In addition, we evaluated the 6 patients (11.8%) who had positive margins after ESD but did not undergo additional gastrectomy. Of the 2 patients (3.9%) who had positive horizontal margins underwent additional ESD, one had cancer but the other did not, and both are alive without recurrence. Of the 4 patients (7.8%) who had positive vertical margins, 2 patients are alive without recurrence, the other one died of liver metastasis of gastric cancer without local RC, and the remaining one died of advanced pancreatic cancer without performing endoscopy.

**Fig. 2:**
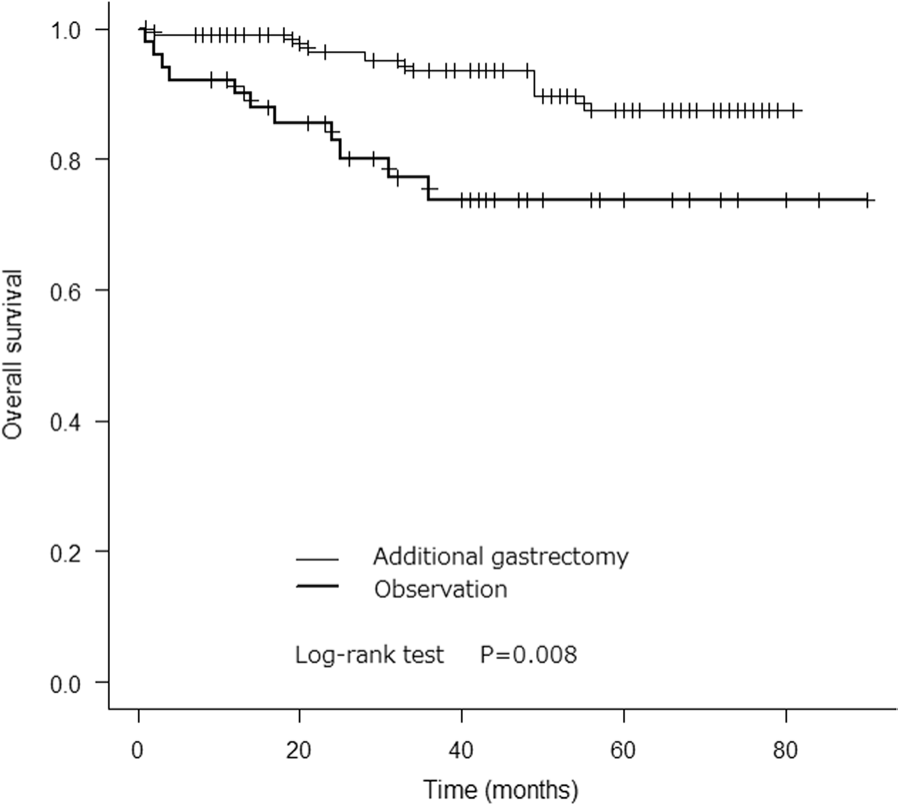
Kaplan–Meier analysis of overall survival rates in the additional gastrectomy and observation groups

**Fig. 3:**
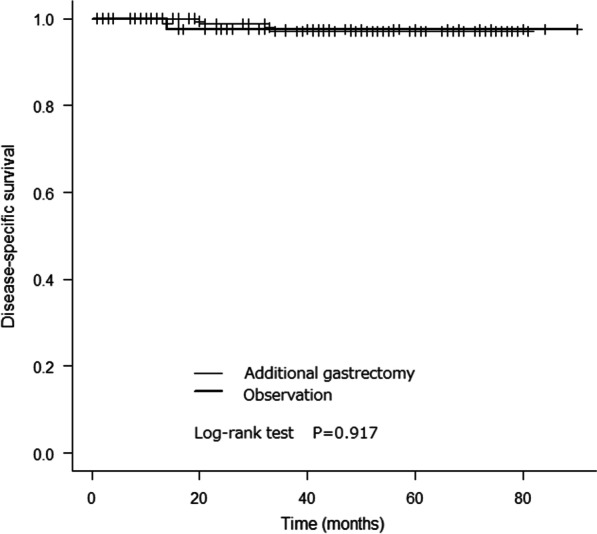
Kaplan–Meier analysis of disease-specific survival rates in the additional gastrectomy and observation groups

The 5-year overall survival rates in the additional gastrectomy and observation groups were 87.4% and 73.8%, respectively (Fig. 2). There was a significant difference between the overall survival rates in the 2 groups (log-rank test, p = 0.008). The 5-year disease-specific survival rates in the 2 groups were 97.2% and 97.6%, respectively (Fig. 3). There was no significant difference between the disease-specific survival rates in the 2 groups (log-rank test, p = 0.917).

## Discussion

Endoscopic submucosal dissection is a minimally invasive and effective procedure for early gastric cancer with a negligible risk of LNM. When the pathological findings do not meet the curative criteria, an additional gastrectomy with lymph node dissection is recommended [1–3]. However, certain patients, such as older adults, may have additional risks with this surgery [6, 7]. Furthermore, an additional surgical resection may amount to overtreatment for approximately 90% or more of the patients identified in ESD with noncurative treatment [8]. Therefore, whether additional surgery should be performed after noncurative ESD for early gastric cancer is controversial [4, 5, 9]. This retrospective analysis revealed that the incidence rate of LNM in patient receiving additional gastrectomy was 14.0% (14/100), which is slightly higher but comparable to incidence rates previously reported by others ( ranging from 6.3% to 12.7%) in studies on additional gastrectomy after ESD [2, 10–14].

Previous reports identified several predictive factors for LNM in cases of early gastric cancer, including a submucosal invasion, a positive lymphatic invasion, a positive vascular invasion, an undifferentiated tumor type, and a tumor size > 30 mm [14–16].

These factors are critical in determining whether to perform an additional gastrectomy with lymph node dissection in patients not meeting the curative criteria after ESD. In this study, lymphatic invasion and undifferentiated type were independent risk factors for LNM in patients not meeting the curative criteria after ESD.

Furthermore, having a vascular invasion was correlated with LNM in a univariate analysis. The rate of LNM was observed in 41.2% (7/17) of patients with both lymphatic.

invasion and vascular invasion. In contrast, the rate of LNM was only observed in 3.8% (2/52) of patients who had neither a lymphatic nor vascular invasion. Of patients not meeting the curative criteria after ESD, those without both lymphatic and vascular invasions are considered to be at low risk of LNM. Therefore, both factors being positive are thought to be a markedly high-risk factor for LNM. Neither the tumor size > 30 mm nor deeper submucosal invasion (SM2) was correlated with LNM in this study. Hatta et al. [15] established the eCura scoring system to predict the risk of LNM in patients after a noncurative ESD. This system evaluates patients on 5 factors: the presence of a lymphatic invasion, tumor size > 30 mm, positive vertical margin, venous invasion, and SM2 invasion. According to this scoring system, patients are categorized into 3 risk groups: low, intermediate, and high. Salvage surgery is expected to benefit those in the high-risk group, while follow-ups alone are sufficient for those in the low risk group [15]. In this system, the presence of two or more other positive factors in addition to positive lymphatic invasion is classified as a high-risk group. When evaluating this system in our study, the incidence rate of LNM was 31.3%(10/32)in the high-risk group, 8.8%(3/34)in the intermediate group, and 2.9% (1/34) in the low risk group (data not shown). The eCura criteria might make it possible to select appropriate patients for additional surgery. On the other hand, caution is needed when applying this system to patients with undifferentiated type [17]. In our study, it seemed that the presence of both positive lymphatic invasion and positive vascular invasion was particularly more important among these 5 factors. Therefore, follow-ups without additional surgery may be sufficient not only for patients of advanced age, or those with severe comorbidities, but also for patients in whom lymphovascular invasion is absent.

In this study, local RC after an additional gastrectomy was observed in 7 patients (7.0%). Multivariate analysis identified as an independent risk factor for local RC after ESD included having a positive horizontal margin. Our result is consistent with previous studies that reported having a positive horizontal margin is a risk factor for local RC, but a positive vertical margin is not [14, 18]. This may be explained by the weaker cautery effect in the horizontal compared to vertical direction [14, 18]. Furthermore, having an undifferentiated tumor type was not significantly related to the risk of local RC in this study. After noncurative ESD, treatment options include additional surgery and additional endoscopic procedures. Because of the low risk of LNM in cases with a positive horizontal margin and a differentiated tumor type without positive lymphatic invasion, local treatment, such as a secondary ESD, can be considered [2, 14, 19]. Confirming the utility of this approach, one of 2 patients in the observation group who had local recurrence underwent secondary ESD and is alive without recurrence. Endoscopic treatment may be possible for patients with positive horizontal margins of the differentiated type without lymphatic invasion because of the low risk of LNM.

The local recurrence rate after an en bloc resection with a negative margin by ESD has been reported to range between 0% and 0.7% [20–22]. For comparison, there were two patients in our study that had local RC observed after en bloc resection with negative margin. One patient was in the additional gastrectomy group, the other in the observation group. Both of these lesions were large tumors with lymphovascular invasion. Previous reports stated that in these cases the cancer cells might have spilled out from the vessels, accumulated within the vessels due to vascular stasis, or remained after resection because the coagulated portion of the resection margin could not be assessed accurately [14, 20].

In this study, a positive lymphatic invasion and a positive vertical invasion, considered high risks of LNM were observed more frequently in the additional gastrectomy group, whereas older patients and higher ASA-PS scores were observed more frequently in the observation group. Furthermore, the 5-year overall survival rate was significantly low in the observation group, and the majority of deaths (90.9%, 10/11) in the observation group died of causes other than gastric cancer during the follow-up period. Although, the 5-year disease-specific survival rates did not differ significantly between the additional gastrectomy and observation groups. Therefore, the physician may have selected observation instead of additional surgical resection, for the patients with older ages or several comorbidities.

We have found that having SM2 or a deeper invasion was not associated with the risk of LNM, but the reverse was not true: all patients with LNM had SM2 or a deeper invasion. Significantly, all three patients who experienced a recurrence after the additional.

gastrectomy had 2 or more LNM. There is no consensus or ideal recommendation for the extent of lymph node dissection in the additional surgery [23]. A D1 + lymph node dissection was performed as standard treatment in our study. However, D2 dissection was selected, when the lesion depth was SM2 with a positive vertical margin or when LNM was clinically suspected. In our study, all LNM instances were within the D1 + level. Therefore, performing a D1 + dissection appears reasonable after noncurative ESD for early gastric cancer.

Surgery related complications classified as CD grade II or more occurred in 14 patients (14.0%) in this study. One patient died due to the postoperative complication. The median age of patients with postoperative complications grade II or more CD classification was 75 years, and older age was significantly related to the occurrence of postoperative complications. Clinicopathological characteristics of the patients did not differ significantly between the additional gastrectomy and observation groups. Although, age and ASA-PS scores were significantly high in the observation group. Therefore, an additional gastrectomy with a lymphadenectomy may be more appropriate than simple follow-ups in noncurative ESD patients without concomitant disease who are young enough to undergo a surgical intervention [1, 24].

This study has some limitations. First, this study was retrospective, and we used the relatively small sample size from a single institute. Second, the criteria of ESD differed slightly depending on doctors who performed the treatment. Third, the average age of the observation group was significantly higher than that of the additional surgery group. There was a selective bias of not undergoing additional surgery due to patients’ old ages, comorbidities, and their own decision.

To address these limitations, a prospective, multicenter, large-scale further analysis should be considered.

## Conclusions

In patients not meeting the curative criteria after ESD for early gastric cancer, a positive lymphatic invasion and an undifferentiated type were independent risk factors for LNM, while a positive horizontal margin was an independent risk factor for local RC. Based on our results, we recommend an additional gastrectomy with lymph node dissection for patients with lymphovascular invasion and/or undifferentiated type after noncurative ESD. Additional surgery under these circumstances has been proven to be effective and to have good long-term outcomes. However, it may be possible to use local treatment, such as a secondary ESD, for patients with a positive horizontal margin without a lymphovascular invasion, and it may be acceptable to carefully follow up without additional surgery for those patients who have no lymphovascular invasion, or are of an advanced age or with a severe comorbidity.

## Data Availability

The datasets used and analyzed during the current study are available from the corresponding author on reasonable request.

## Abbreviations

ESD: Endoscopic submucosal dissection
LNM: : Lymph node metastasis
RC: : Residual cancer
M: : Mucosal
SM: : Submucosal
HM0: : Negative horizontal margin
VM0: : Negative vertical margin
CT: : Computed tomography
CD: : Clavien-Dindo
ASA-PS: : American Society of Anesthesiologists physical status
IQR: : Interquartile range
SSI: : Surgical site infection
